# Efficacy of Compound Herbal Medicine Tong-Xie-Yao-Fang for Acute Radiation Enteritis and Its Potential Mechanisms: Evidence from Transcriptome Analysis

**DOI:** 10.1155/2020/5481653

**Published:** 2020-12-01

**Authors:** Cheng Yang, Jiayue Yang, Min Xia, Jianhong Wei, Yang Jiao, Qiang Zhan

**Affiliations:** ^1^Department of Gastroenterology, Wuxi People's Hospital Affiliated to Nanjing Medical University, Wuxi, 214023 Jiangsu Province, China; ^2^Department of Endocrinology, Wuxi People's Hospital Affiliated to Nanjing Medical University, Wuxi, 214023 Jiangsu Province, China; ^3^School for Radiological and Interdisciplinary sciences, Soochow University, Suzhou, 215123 Jiangsu Province, China

## Abstract

Acute radiation enteritis (ARE) is a common complication with radiotherapy for pelvic and abdominal malignancy. This research is designed to investigate the efficacy of Tong-Xie-Yao-Fang (TXYF) on ARE and to explore the underlying mechanisms by microarray analysis. The ARE rat model was established by a single abdominal irradiation with a gamma-ray dose of 10 Gy. Next, the ARE rats were treated with distilled water, TXYF, and glutamine by gavage for 7 consecutive days according to the scheduled groups. For each group, the jejunal tissue was taken at 6 h after gastric lavage. The morphology of intestinal tissue was observed by hematoxylin and eosin (H&E) stain under a light microscope. The height of the villus and the thickness of the whole layer of the TXYF-treated groups were significantly ameliorative than that of the model control group. The transcriptome analysis was produced using the Agilent SurePrint G3 Rat GE V2.0 microarray. A total of 90 differentially expressed genes (DEGs), including 48 upregulated genes and 42 downregulated genes, were identified by microarray and bioinformatics analysis. Protein–protein interaction (PPI), Gene Ontology (GO), and Kyoto Encyclopedia of Genes and Genomes (KEGG) were conducted to explore the possible mechanisms of DEGs taking part in the TXYF-mediated therapeutic process for ARE. In conclusion, we reveal that TXYF has a protective effect on the intestinal tissue of rats with ARE and summarize several DEGs, suggesting the possible mechanisms of TXYF-mediated efficacy for ARE.

## 1. Introduction

In recent years, the clinical consensus for radiation therapy of tumors has been reached, and treatment is standardized gradually [1, 2]. However, with the growing incidence of tumors and the increasing popularity of radiotherapy, increasing numbers of patients inevitably develop acute radiation enteritis (ARE) after radiation therapy for pelvic and abdominal malignancies [3]. ARE is a common intestinal complication during and after radiotherapy for abdominal and pelvic malignancies. Clinical manifestations of ARE commonly include abdominal pain, diarrhea, bloody stool, even sepsis, systemic inflammation, and multiple organ dysfunction syndromes, threatening patients' lives [4, 5]. However, there is no standardized treatment for ARE, and symptomatic support is the main treatment at present.

Although western medicine treatment can achieve certain effects in clinical practice, the overall effect is still not satisfactory. As one of China's traditional medical treasure houses, traditional Chinese medicine has unique advantages in the treatment of gastrointestinal diseases [6–8]. Tong-Xie-Yao-Fang (TXYF) is one of the classic prescriptions of traditional Chinese medicine, which consists of four herbal drugs: *Rhizoma Atractylodis Macrocephalae*, *Radix Paeoniae Alba*, *Pericarpium Citri Reticulatae*, and *Radix Saposhnikoviae*. This prescription has been widely applied to clinical treatment for gastrointestinal diseases in China, including rectal ulcer syndrome and irritable bowel syndrome [9–11]. Li et al. applied the UPLC-MS/MS method to identify eleven bioactive components of TXYF, including 1 lactone, 2 monoterpene glucosides, 1 alkaloid, 5 flavonoids, and 2 chromones, revealing the pharmacological mechanism of TXYF [12]. However, whether TXYF has efficacy on ARE and its potential mechanisms remain largely unclear.

In this study, we established a physiologically relevant ARE rat model and observed the efficacy of TXYF on ARE by evaluating the pathological morphology of the small intestinal mucosa after irradiation and treatment. We further explore the potential mechanisms of TXYF treating ARE based on transcriptome analysis and bioinformatics analysis.

## 2. Methods and Materials

### 2.1. Reagents and Instruments

TXYF was prepared with *Rhizoma Atractylodis Macrocephalae*, *Radix Paeoniae Alba*, *Pericarpium Citri Reticulatae*, and *Radix Saposhnikoviae*, which were purchased from the Wuxi Hospital of Traditional Chinese Medicine and composed in 6 : 4 : 3 : 2 proportions. Raw components were soaked in a 10 times volume of distilled water for 0.5 h and boiled twice, first for 1.5 h and then for 1 h. Two of the boiled ingredients were filtered, mixed together, and concentrated in a 1 : 1 ratio (100% concentration) and stored at 4°C for later use. TXYF was diluted in distilled water to a concentration of 4.92 g/mL and stored at room temperature before use.

The following reagents were used: glutamine (ST083; Beyotime Biotechnology, China), PBS buffer (C0221A; Beyotime Biotechnology, China), and distilled water (GB19298; Watsons, China). The following instruments were used: linear accelerator (Elekta, Sweden), light microscope (Nikon, Japan), and histotome (LEICA, Germany).

### 2.2. Animal Model

Forty-eight Sprague-Dawley (SD) male rats weighing 200-220 g (NO.201805475) were purchased from Changzhou Cavens Animal Co. Ltd (Changzhou, China). All rats were housed in Animal Experiment Center of Wuxi People's Hospital (SYXK(SU)2015-0004) maintained at constant temperature and humidity with a 12/12 h light/dark cycle according to the guidelines established by the Animal Core Facility of Nanjing Medical University. A total of 48 SD rats were randomly divided into four groups: A-D. Group A (*n* = 12) was given no treatment, while group B (*n* = 12), group C (*n* = 12), and group D (*n* = 12) underwent the whole abdominal irradiation at a single dose of 10 Gy. On the day 1 after irradiation, group A and group B were given distilled water, while group C was given TXYF (4.92 g/100 g) and group D were given glutamine (0.3 g/100 g) by gavage for 7 consecutive days. The volume of medicine was 2 mL/100 g/d, and the same volume of distilled water was given to groups A and B. The rats were euthanized by an excessive overdose of anesthetic sodium pentobarbital injection. For each group, the jejunal tissue was taken at 6 h after gastric lavage, and the morphology of intestinal tissue was observed by hematoxylin and eosin (H&E) staining (KenGEN BioTECH, Nanjing, China) under a light microscope. Three sections extracted from each samples were checked. The sections were independently evaluated by two pathologists. All experimental procedures were approved by the Supervisory Committee of Nanjing Medical University Animal Council.

### 2.3. RNA Extraction and Microarray Scanning

Three samples were extracted randomly from groups B and C, respectively. Total RNA was extracted from these six jejunal tissue samples by using the mirVana™ isolation kit (Ambion, Austin, TX, USA), quantified by the NanoDrop ND-2000 (Thermo Scientific), and the RNA integrity was assessed using Agilent Bioanalyzer 2100 (Agilent Technologies). The sample labeling, microarray hybridization, and washing were performed based on the manufacturer's standard protocols. Briefly, total RNA was transcribed to double-strand cDNA, then synthesized into cRNA, and labeled with Cyanine-3-CTP (OE BioTECH, Shanghai, China). The labeled cRNAs were hybridized onto the Agilent SurePrint G3 Rat GE V2.0 microarray. After washing, the arrays were scanned by the Agilent Scanner G2505C (Agilent Technologies).

### 2.4. Microarray Data Analysis

Feature extraction (version10.7.1.1, Agilent Technologies) was used to analyze array images to get raw data. GeneSpring (version13.1, Agilent Technologies) was employed to finish the basic analysis with the raw data. To begin with, the raw data were normalized with the quantile algorithm. The probes that at least 100% of the values in any 1 out of all conditions have flags in “Detected” were chosen for further data analysis. Differential probes were then identified through fold change (FC) as well as *P* value calculated with *t*-test. The threshold set for up- and downregulated genes was the FC ≥ 1.5 and the *P* value ≤ 0.05. Afterward, hierarchical clustering was performed to display the distinguishable probes' expression pattern among samples. Finally, Gene Ontology (GO) and Kyoto Encyclopedia of Genes and Genomes (KEGG) analysis were applied to determine the roles of differentially expressed genes (DEGs) according to differential probes.

### 2.5. Protein–Protein Interaction (PPI) Network Construction

The protein–protein interaction (PPI) network was constructed using the STRING database (https://string-db.org/) to load all the DEGs [13]. For all other parameters, the default settings were used. ∗.tsv format network files were loaded into the plug-in cytoHubba based on the Cytoscape software [14]. We defined the top 5 genes with the highest prediction scores calculated by the MCC algorithm. Besides, the network diagram of PPI was visualized with Cytoscape.

### 2.6. Statistical Analysis

All statistical analyses were performed on IBM SPSS Statistics 25.0. Most of the data were analyzed by Student's *t*-test or one-way ANOVA followed by Tukey's test. All data are presented as means ± SDs of five independent experiments if not noted. For all analyses, differences were considered statistically significant if *P* values were less than 0.05.

## 3. Results

### 3.1. Establishment of the Rat Model for Irradiation-Induced Acute Radiation Enteritis

**Figure 1:**
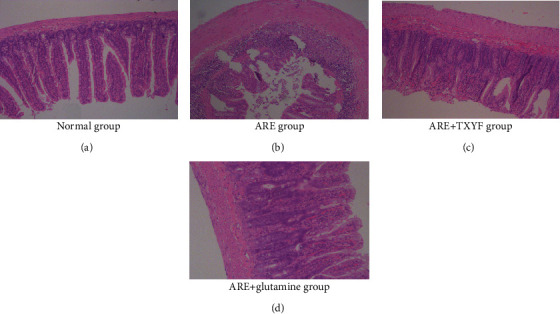
Difference in bowel morphology between four groups. The morphology (×100) of intestinal tissue was observed by hematoxylin and eosin (H&E) stain under a light microscope after seven days of irradiation. (a) Normal group; (b) acute radiation enteritis group; (c) ARE rats treated with TXYF group; and (d) ARE rats treated with glutamine group. Bar = 100 *μ*m.

**Figure 2:**
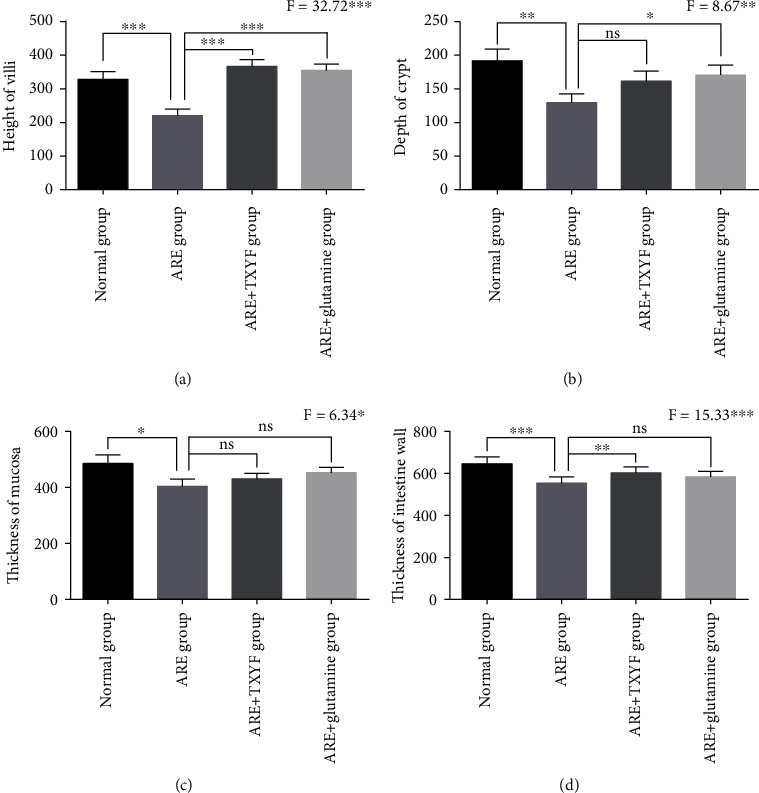
Quantitative analysis of bowel morphology changes between four groups. Bowel morphology changes of forty-eight rats were analyzed by quantitative count. (a) Height of villus; (b) crypt depth; (c) mucosal thickness; and (d) intestine wall thickness.

There was no significant difference in the mental state and food intake of the rats in each group before irradiation. On the day 2 after irradiation, the rats in groups B, C, and D had worse mental states and less food intake than those in group A. Besides, ARE rats showed obvious diarrhea, while the control rats continued to defecate normally. For each group, the jejunal tissue was taken at 6 h after last gastric lavage, and H&E staining revealed dramatic destruction in the intestinal of irradiated animals, whereas the intestinal structure of control rats kept normal (Figures 1(b) and 1(c)). Further quantitative analysis confirmed the successful establishment of the ARE rat model. Compared with control rats, ARE rats receiving no treatments exhibited significant decreasing in villus height, crypt depth, mucosa, and full thickness (Figures 2(a)–2(d)). All these results confirmed the occurrence of ARE in rats from group B.

### 3.2. Efficacy of Tong-Xie-Yao-Fang on Acute Radiation Enteritis

As we described before, on the day 2 after irradiation, the rats in groups B, C, and D had worse mental states and less food intake than compared with group A rats. On the day 1 after irradiation, group A and group B were given distilled water, while group C was given TXYF and group D was given glutamine by gavage for 7 consecutive days. Glutamine is an effective drug for ARE in both clinical practice and laboratory according to previous publications [15, 16]. On the day 7 after irradiation, the above symptoms of the rats in group B still existed and no improvement was exhibited; moreover, one rat died on day 3. However, the mental status and the response to the outside of rats from groups C and D were improved, and food intake was increased. H&E staining and quantitative analysis confirmed significant increase in the villus height and the full thickness in TXYF-treated rats, although depth of crypt and thickness of mucosa had no notable difference (Figures 1(b), 1(c), and 2(a)–2(d)). To conclude, these findings suggested that TXYF has obvious efficacy on ARE *in vivo*.

### 3.3. Quality Control of Microarray Analysis

**Figure 3:**
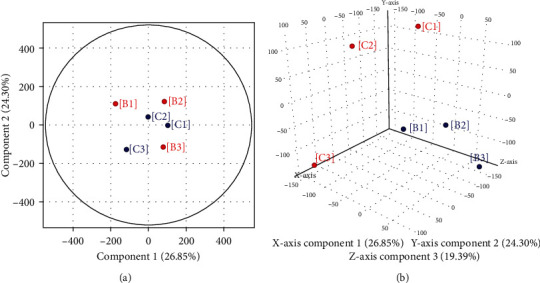
PCA score plots based on the microarray data. The distribution of DEGs for the samples from groups B and C in PCA. (a) The distribution in two-dimensional space. (b) The distribution in three-dimensional space.

To explore the potential mechanism of TXYF-mediated efficacy for ARE, we next performed transcriptome analysis of jejunal tissues from groups B and C. Before the subsequent analysis, we applied the relative logarithmic expression (RLE) boxplots and principal component analysis (PCA) to control the quality of the microarray data. RLE boxplots revealed the symmetry of the data was good, suggesting the quality of total RNA was reliable (Figure S1). Through PCA analysis, the distribution of samples was examined to verify the rationality of the experimental design and the uniformity of biological duplicate samples. As shown in Figure 3, samples from the same group are distributed closely in the two-dimensional (Figure 3(a)) or three-dimensional space (Figure 3(b)), suggesting samples involved in this research were representative and biological repetitive.

### 3.4. Identification of Differentially Expressed Genes

**Figure 4:**
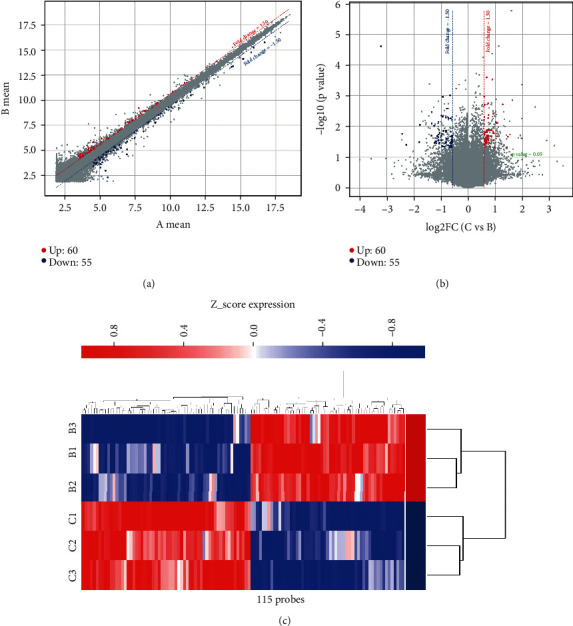
Heatmap of clustering analysis of ARE rats and normal controls. (a) The raw data is normalized and converted into log base 2 logarithms, which are represented in a two-dimensional rectangular coordinate system plane as a scatter plot. (b) *t* test was used to analyze the differential probes which represented as a volcano plot. (c) Cluster assay was used to analyze the expression of 115 differential probes in normal and ARE groups.

DEGs were applied to Student's *t*-test at univariate to check the differential expression levels. We totally identified 115 differential probes with the FC ≥ 1.5 and the *P* value ≤ 0.05 as potential candidates accounting for TXYF-induced efficacy (Figures 4(a) and 4(b)). The heatmap of hierarchical clustering analysis was a useful tool to reveal the expression differences of differential probes intuitively. We can see the similarity of the differential probes abundance profiles (Figure 4(c)), exhibiting a satisfactory discriminatory value between the two groups. A total of 115 differential probes corresponded to 90 functional DEGs, including 48 upregulated probes and 42 downregulated probes. The list of 90 DEGs was shown in Table S1.

### 3.5. Protein–Protein Network Construction and Enrichment Analysis

**Figure 5:**
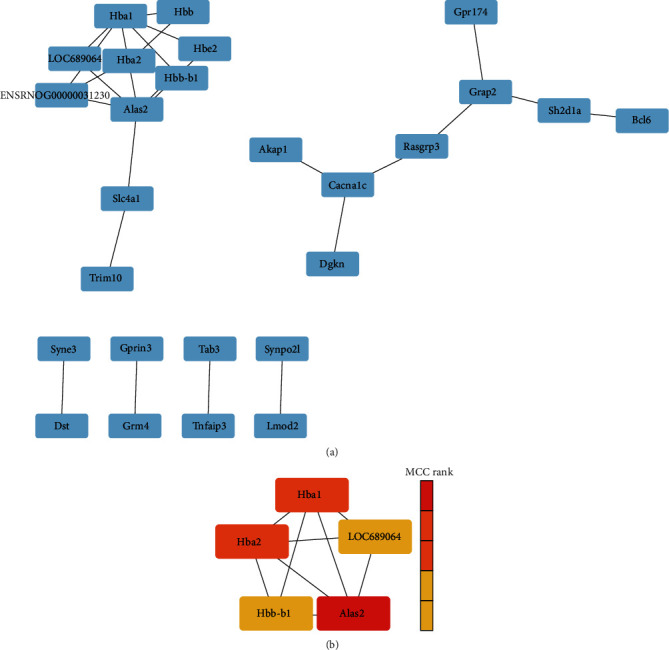
PPI network construction of DEGs. (a) PPI network of DEGs in the TXYF-treated group. (b) The genes with the top 5 prediction scores calculated with the MCC algorithm.

We searched those 90 DEGs in STRING and visualized the network using Cytoscape (Figure 5(a)). Based on the MCC algorithm, we extracted the top five genes (*Alas2*, *Hba1*, *Hba2*, *LOC689064*, and *Hbb-b1*) showing the closest connections with other genes, and *Alas2* showed the closest connections (Figure 5(b)).

**Figure 6:**
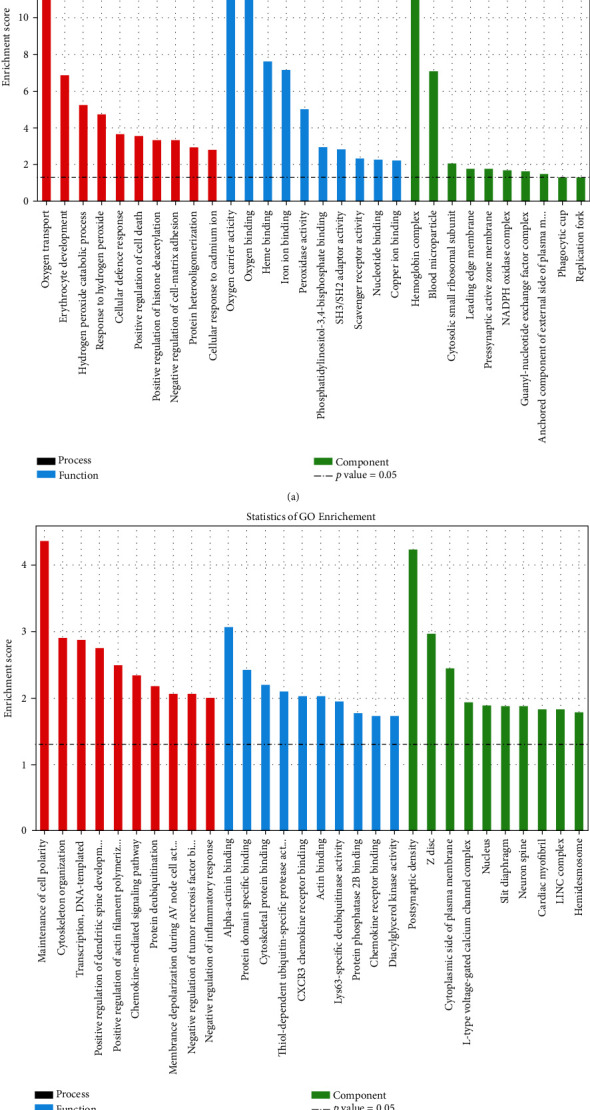
GO analysis of DEGs between groups C and D. GO enrichment analysis predicted the functional roles of target host genes based on three aspects. (a) Upregulated genes. (b) Downregulated genes. Top 10 terms of BP, CC, and MF analyses are represented in this figure. Red: BP; blue: MF; green: CC.

**Figure 7:**
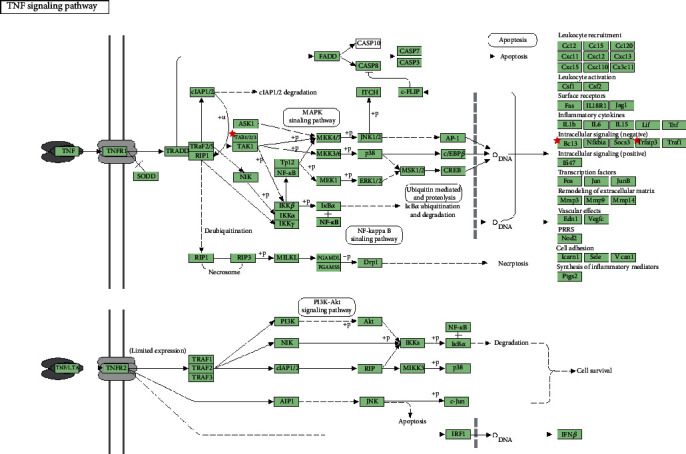
An overview of TNF pathway. Tab3, Bcl3, and Tnfaip3 were downregulated and had potential inhibited effects on TNF pathway.

GO enrichment analysis was next performed to predict the functional roles of DEGs based on three aspects including biological processes (BP), molecular functions (MF), and cellular components (CC). Several functional roles of both upregulated genes (Figure 6(a), Table S2) and downregulated genes (Figure 6(b), Table S3) were uncovered. With the KEGG enrichment analysis, a total of eight pathways were identified, including three pathways related to upregulated genes and five DEG pathways related to downregulated genes (Table 1). Among all pathways, the top-ranking enriched terms were TNF signaling pathway for downregulated genes, which is mostly associated with inflammation among all pathways (Figure 7).

## 4. Discussion

The mechanism of ARE occurrence is very complicated. Modern medicine holds the opinion that ARE is mainly caused by reduced mitosis of mucosal crypts, destruction of the intestinal mucosal barrier, and acute inflammation [17]. The intestinal epithelial tissue is renewed quickly, and it is renewed every 3-5 days, and the sensitivity of human tissues to radioactivity is proportional to its proliferative capacity [18]. Therefore, the rapidly proliferating intestinal epithelium is more sensitive to ionizing radiation and the risk of damage is also relatively large. Radiation could cause the mitosis of stem cells in the intestinal crypts to be inhibited, or even stopped, and degeneration and necrosis occur, interrupting the supply of cells to the villi, shortening the villi to bareness, and even destroying the structural integrity of the intestinal mucosa [19]. With the popularity of radiation therapy for tumors, the incidence of ARE is increasing largely. However, no standardized treatment has been established for ARE in clinical practice up to date.

In this study, we established a physiologically relevant ARE rat model. The jejunal mucosal villous was obvious edema, accompanied by partial villous epithelial cells falling off and decreased crypt depth and inflammatory cell infiltration, indicating the successful establishment of ARE model. After the application of TXYF, the villi were more complete, the mucosal edema was lighter, and the height of villi and crypt depth were increased in experimental rats, which significantly improved than those in the control group. Glutamine is an effective drug for ARE; encouragingly, no notable difference in bowel morphology was observed between the TXYF and glutamine group. It is suggested that TXYF could reduce tissue damage and accelerate intestinal repair, showing promising efficacy for ARE *in vivo*.

In the past decades, vigorously developing high-throughput sequencing technology and computer-aided analytical methods have largely promoted the flourish of big data applications [20–22]. As an effective research strategy, transcriptome analysis has been widely used in several aspects of clinical or basic medical research [23]. Due to the multiple active ingredients of compound Chinese medicine, the mechanism of action tends to be complex. At present, transcriptome analysis has been applied to uncover mechanisms of compound Chinese medicine in clinical practice [24].

To explore the potential mechanism of TXYF in treating ARE, six samples extracted from ARE rats and treated with TXYF rats were submitted into transcriptome analysis. Finally, we totally identified 90 DEGs, and we next conducted the PPI network construction and enrichment analysis with the DEGs by GO and KEGG pathway analysis to get a better view of the overall DEGs in TXYF-treated tissues. As the result showed, *Alas2* exhibited the closest connections with other genes. It has been shown that growth hormone could increase *Alas2* gene expression in the rat brain [25, 26]. However, the roles of Alas2 in ARE have not been defined.

GO enrichment analysis predicted the functional roles of both upregulated and downregulated DEGs. With the KEGG enrichment analysis, eight significant pathways were identified. Among all pathways, top-ranking enriched terms were Malaria and TNF signaling pathway for upregulated and downregulated genes, respectively. As one of inflammation-associated pathways, the roles of TNF signaling pathway in ARE have not been investigated. As we all know, inhibition of TNF pathways could suppress inflammation, and TXYF treatment downregulated *Tab3*, *Bcl3*, and *Tnfaip3* gene expression, inhibiting TNF pathway to some extent. Overall, further research should be performed to explore the potential mechanisms of TXYF treating ARE.

## 5. Conclusion

In summary, we observe that TXYF has promising efficacy on ARE. Further research reveal several DEGs in the jejunal tissues in response to TXYF treatment, suggesting the possible mechanisms of TXYF-mediated efficacy for ARE. We hope to establish the theoretical basis of TXYF-based treatment for radiation enteritis.

## Figures and Tables

**Table 1:** KEGG analysis of dysregulated genes in ARE and ARE+TXYF groups.

Term_ID	Term_description	*P* value
Upregulated genes		
path:rno05144	Malaria	1.06E-10
path:rno05143	African trypanosomiasis	4.82E-10
path:rno00860	Porphyrin and chlorophyll metabolism	0.005
Downregulated genes		
path:rno04668	TNF signaling pathway	2.40E-04
path:rno04657	IL-17 signaling pathway	0.005
path:rno04064	NF-kappa B signaling pathway	0.006
path:rno04621	NOD-like receptor signaling pathway	0.016
path:rno04614	Renin-angiotensin system	0.047

## Data Availability

Data will be provided on request through the corresponding author of this article.

## Supplementary Materials

Supplementary Materials Figure S1: relative logarithmic expression (RLE) boxplots for six samples. Table S1: the list of 90 DEGs. Table S2: GO enrichment analysis terms of upregulated DEGs. Table S3: GO enrichment analysis terms of downregulated DEGs.

## References

[1] Lim K., Small W., Portelance L. (2011). Consensus guidelines for delineation of clinical target volume for intensity-modulated pelvic radiotherapy for the definitive treatment of cervix cancer. *International Journal of Radiation Oncology • Biology • Physics*.

[2] Wu A. J., Bosch W. R., Chang D. T. (2015). Expert consensus contouring guidelines for intensity modulated radiation therapy in esophageal and gastroesophageal junction cancer. *International Journal of Radiation Oncology • Biology • Physics*.

[3] Shadad A. K., Sullivan F. J., Martin J. D., Egan L. J. (2013). Gastrointestinal radiation injury: prevention and treatment. *World Journal of Gastroenterology*.

[4] Huang Y., Guo F., Yao D., Li Y., Li J. (2016). Surgery for chronic radiation enteritis: outcome and risk factors. *The Journal of Surgical Research*.

[5] Grabenbauer G. G., Holger G. (2016). Management of radiation and chemotherapy related acute toxicity in gastrointestinal cancer. *Best Practice & Research. Clinical Gastroenterology*.

[6] Teschke R., Wolff A., Frenzel C., Eickhoff A., Schulze J. (2015). Herbal traditional Chinese medicine and its evidence base in gastrointestinal disorders. *World Journal of Gastroenterology*.

[7] Bi Z., Zheng Y., Yuan J., Bian Z. (2017). The efficacy and potential mechanisms of Chinese herbal medicine on irritable bowel syndrome. *Current Pharmaceutical Design*.

[8] Hwang M. W., Ahn T. S., Hong N. R. (2015). Effects of traditional Chinese herbal medicine San-Huang-Xie-Xin-Tang on gastrointestinal motility in mice. *World Journal of Gastroenterology*.

[9] Zhao X. Y., Wang J. W., Yin Y., Li K., Zhang M., Yan F. P. (2019). Effect of Tong Xie Yao Fang on endogenous metabolites in urine of irritable bowel syndrome model rats. *World Journal of Gastroenterology*.

[10] Zhang L. L., Hao W. S., Xu M., Li C., Shi Y. Y. (2019). Modified Tong Xie Yao Fang relieves solitary rectal ulcer syndrome: a case report. *World Journal of Clinical Cases*.

[11] Ma X., Wang X., Kang N. (2017). The effect of Tong-Xie-Yao-Fang on intestinal mucosal mast cells in postinfectious irritable bowel syndrome rats. *Evidence-based Complementary and Alternative Medicine*.

[12] Li T. X., Hu L., Zhang M. M. (2014). A sensitive UPLC-MS/MS method for simultaneous determination of eleven bioactive components of Tong-Xie-Yao-Fang decoction in rat biological matrices. *Journal of Chromatography. B, Analytical Technologies in the Biomedical and Life Sciences*.

[13] Szklarczyk D., Franceschini A., Wyder S. (2015). STRING v10: protein-protein interaction networks, integrated over the tree of life. *Nucleic Acids Research*.

[14] Chin C. H., Chen S. H., Wu H. H., Ho C. W., Ko M. T., Lin C. Y. (2014). cytoHubba: identifying hub objects and sub-networks from complex interactome. *BMC Systems Biology*.

[15] Vidal-Casariego A., Calleja-Fernández A., de Urbina-González J. J. O., Cano-Rodríguez I., Cordido F., Ballesteros-Pomar M. D. (2014). Efficacy of glutamine in the prevention of acute radiation enteritis: a randomized controlled trial. *Journal of Parenteral and Enteral Nutrition*.

[16] Cao D. D., Xu H. L., Xu M., Qian X. Y., Yin Z. C., Ge W. (2017). Therapeutic role of glutamine in management of radiation enteritis: a meta-analysis of 13 randomized controlled trials. *Oncotarget*.

[17] Shadad A. K., Sullivan F. J., Martin J. D., Egan L. J. (2013). Gastrointestinal radiation injury: symptoms, risk factors and mechanisms. *World Journal of Gastroenterology*.

[18] Rath E., Moschetta A., Haller D. (2018). Mitochondrial function - gatekeeper of intestinal epithelial cell homeostasis. *Nature Reviews Gastroenterology & Hepatology*.

[19] Moussa L., Usunier B., Demarquay C. (2016). Bowel radiation injury: complexity of the pathophysiology and promises of cell and tissue engineering. *Cell Transplantation*.

[20] Tang R. X., Chen W. J., He R. Q. (2017). Identification of a RNA-Seq based prognostic signature with five lncRNAs for lung squamous cell carcinoma. *Oncotarget*.

[21] Cai Y., Mei J., Xiao Z. (2019). Identification of five hub genes as monitoring biomarkers for breast cancer metastasis in silico. *Hereditas*.

[22] Chen J., Cai Y., Xu R., Pan J., Zhou J., Mei J. (2020). Identification of four hub genes as promising biomarkers to evaluate the prognosis of ovarian cancer in silico. *Cancer Cell International*.

[23] Casamassimi A., Federico A., Rienzo M., Esposito S., Ciccodicola A. (2017). Transcriptome profiling in human diseases: new advances and perspectives. *International Journal of Molecular Sciences*.

[24] Xin J., Zhang R. C., Wang L., Zhang Y. Q. (2017). Researches on transcriptome sequencing in the study of traditional Chinese medicine. *Evidence-based Complementary and Alternative Medicine*.

[25] Walser M., Oscarsson J., Aberg M. A. I., Svensson J., Isgaard J., Aberg N. D. (2019). Effects of peripheral administration of GH and IGF-I on gene expression in the hippocampus of hypophysectomised rats. *Neuro Endocrinology Letters*.

[26] Walser M., Schiöler L., Oscarsson J. (2017). Mode of GH administration and gene expression in the female rat brain. *The Journal of Endocrinology*.

