# Supplementary material for: Efficacy of Compound Herbal Medicine Tong-Xie-Yao-Fang for Acute Radiation Enteritis and Its Potential Mechanisms: Evidence from Transcriptome Analysis
Source: Biomed Res Int. 2020 Dec 1;2020:5481653. doi: 10.1155/2020/5481653 (PMC7725573; doi:10.1155/2020/5481653)
Supplement: Supplementary Materials — Figure S1: relative logarithmic expression (RLE) boxplots for six samples. Table S1: the list of 90 DEGs. Table S2: GO enrichment analysis terms of upregulated DEGs. Table S3: GO enrichment analysis terms of downregulated DEGs. [file 5481653.f1.pdf]

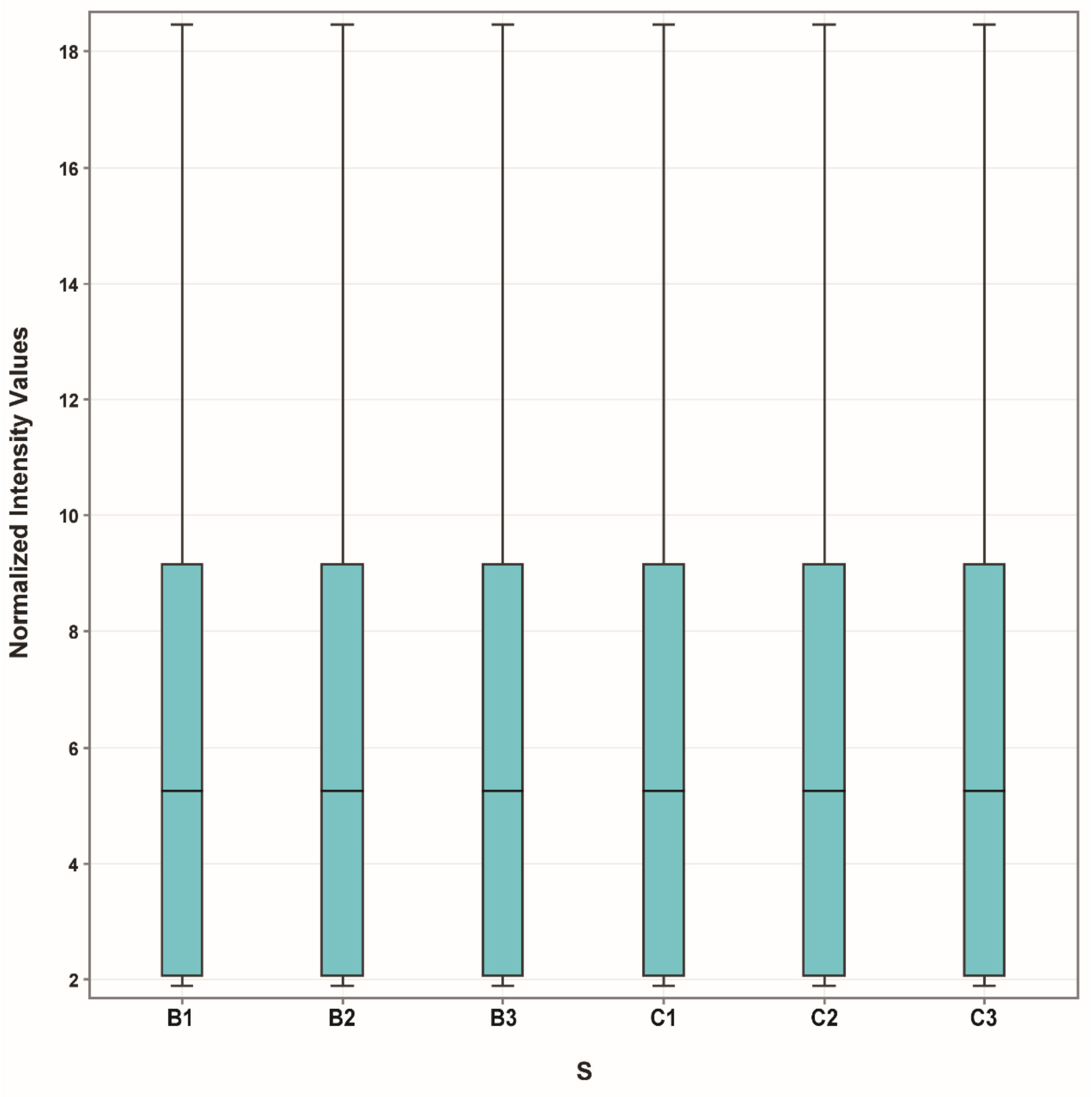

Figure S1. Relative logarithmic expression (RLE) boxplots for six samples

Descriptions of Tab S1-3.

Table S1. The list of 90 DEGs.

Table S2. GO enrichment analysis terms of upregulated DEGs.

Table S3. GO enrichment analysis terms of downregulated DEGs.

GeneSymbol	GeneName	EntrezGeneID	Regulation(C_vs._B)
Akap1	A-kinase anchoring protein 1	114124	down
Alas2	5'-aminolevulinate synthase 2	25748	up
Arhgap26	Rho GTPase activating protein 26	307459	down
Asic4	acid sensing ion channel subunit family memb	63882	down
Bcl3	B-cell CLL/lymphoma 3	680611	down
Bcl6	B-cell CLL/lymphoma 6	303836	up
Bhlhb9	basic helix-loop-helix family member B9	317407	up
Bmx	BMX non-receptor tyrosine kinase	367786	up
Cacna1c	calcium voltage-gated channel subunit alpha1	24239	down
Cdkl3	cyclin-dependent kinase-like 3	60396	up
Celf6	CUGBP, Elav-like family member 6	300758	down
Chd1l	chromodomain helicase DNA binding protein	310707	up
Chi3l1	chitinase 3 like 1	89824	up
Col9a3	collagen type IX alpha 3 chain	362285	up
Cp	ceruloplasmin	24268	up
Cxcl11	C-X-C motif chemokine ligand 11	305236	down
Dgkh	diacylglycerol kinase, eta	361076	down
Dlg5	discs large MAGUK scaffold protein 5	305645	down
Dst	dystonin	316313	down
Enpp2	ectonucleotide pyrophosphatase/phosphodiest	84050	up
Erp27	endoplasmic reticulum protein 27	297698	up
Fam180a	family with sequence similarity 180, member	362336	up
Fam199x	family with sequence similarity 199, X-linked	679168	down
Fcrl2	Fc receptor-like 2	310694	up
Gpr174	G protein-coupled receptor 174	302373	up
Gprin3	GPRIN family member 3	502784	down
Grap2	GRB2-related adaptor protein 2	366962	down
Grm4	glutamate metabotropic receptor 4	24417	up
Gypa	glycophorin A	688972	up
Hba1	hemoglobin, alpha 1	25632	up
Hba2	hemoglobin, alpha 2	360504	up
Hbb	hemoglobin subunit beta	24440	up
Hbb-b1	hemoglobin, beta adult major chain	361619	up
Hbe2	hemoglobin, epsilon 2	502359	up
Hira	histone cell cycle regulator	363849	down
Hunk	hormonally upregulated Neu-associated kinas	288275	down
Klk1c8	kallikrein 1-related peptidase C8	292866	down
Krtap7-1	keratin associated protein 7-1	363741	down
Ldhc	lactate dehydrogenase C	29634	up
Lmod2	leiomodin 2	296935	down
LOC10013487	beta globin minor gene	100134871	up
LOC10090980	uncharacterized LOC100909803	100909803	up
LOC10091126	uncharacterized LOC100911266	100911266	up
LOC10091262	uncharacterized LOC100912622	100912622	down
LOC10254801	uncharacterized LOC102548015	102548015	up
LOC10254917	ankyrin repeat domain-containing protein 26-l	102549174	up
LOC10254928	uncharacterized LOC102549287	102549287	up
LOC10255049	uncharacterized LOC102550495	102550495	up
LOC10369097	uncharacterized LOC103690979	103690979	down

LOC10369190!	uncharacterized LOC103691905	103691905	down
LOC10369263	uncharacterized LOC103692634	103692634	down
LOC10834934	disks large homolog 5-like	108349347	up
LOC10835187	endogenous retrovirus group K member 9 Pol	108351873	down
LOC689064	beta-globin	689064	up
Lpin1	lipin 1	313977	up
Ly49si2	immunoreceptor Ly49si2	494207	up
Mob3c	MOB kinase activator 3C	313511	down
Myo1g	myosin IG	289785	up
Ncf1	neutrophil cytosolic factor 1	114553	up
Nphs1	NPHS1 nephrin	64563	down
Nxpe1	neurexophilin and PC-esterase domain family,	500992	up
Olr59	olfactory receptor 59	170816	up
Opn4	opsin 4	192223	up
Palm	paralemmin	170673	down
Pbx2	PBX homeobox 2	406164	down
Pcdha3	protocadherin alpha 3	116780	up
Prnd	prion like protein doppel	113910	up
Rasgrp3	RAS guanyl releasing protein 3	313874	up
Rnf223	ring finger protein 223	680874	down
Sgpp2	sphingosine-1-phosphate phosphatase 2	301543	down
Sh2d1a	SH2 domain containing 1A	501502	up
Slc4a1	solute carrier family 4 member 1	24779	up
Sorbs2	sorbin and SH3 domain containing 2	114901	down
Stfa2l2	stefin A2-like 2	498087	up
Sult1a1	sulfotransferase family 1A member 1	83783	up
Syne3	spectrin repeat containing, nuclear envelope fa	299356	down
Synpo2l	synaptopodin 2-like	305675	down
Tab3	TGF-beta activated kinase 1 and MAP3K7 bir	317546	down
Tff2	trefoil factor 2	116592	down
Tmed6	transmembrane p24 trafficking protein 6	291991	up
Tmem235	transmembrane protein 235	688285	up
Tnfaip3	TNF alpha induced protein 3	683206	down
Trim15	tripartite motif containing 15	100910806	down
Uhrf1	ubiquitin-like with PHD and ring finger doma	316129	down
Usp35	ubiquitin specific peptidase 35	308834	down
Zbtb16	zinc finger and BTB domain containing 16	353227	up
Zfp37	zinc finger protein 37	115768	down
Zhx3	zinc fingers and homeoboxes 3	311604	down
Znf768	zinc finger protein 768	102553763	down
Zscan10	zinc finger and SCAN domain containing 10	302962	down

Term_ID	Term_description	GeneSymbols	P_value	Category
GO:0005833	hemoglobin complex	Hbe2;LOC689064;Hbb-b1;LC	1.05E-16	CC
GO:0072562	blood microparticle	LOC689064;Hbb;Cp;Hba-a2;1	7.76E-08	CC
GO:0022627	cytosolic small ribosomal sub	Hba1;Hba-a2	0.00814502	CC
GO:0031256	leading edge membrane	Myo1g	0.01565143	CC
GO:0048787	presynaptic active zone memb	Grm4	0.01565143	CC
GO:0043020	NADPH oxidase complex	Ncf1	0.01952692	CC
GO:0032045	guanyl-nucleotide exchange fa	Rasgrp3	0.0214591	CC
GO:0031362	anchored component of extern	Prnd	0.0310646	CC
GO:0001891	phagocytic cup	Myo1g	0.04624302	CC
GO:0005657	replication fork	Bcl6	0.04624302	CC
GO:0005344	oxygen carrier activity	Hbe2;LOC689064;Hbb-b1;LC	6.86E-16	MF
GO:0019825	oxygen binding	Hbe2;LOC689064;Hbb-b1;LC	3.89E-14	MF
GO:0020037	heme binding	Hbe2;LOC689064;Hbb-b1;LC	2.30E-08	MF
GO:0005506	iron ion binding	Hbe2;LOC689064;Hbb-b1;LC	6.97E-08	MF
GO:0004601	peroxidase activity	Hba1;Hbb;Hba-a2	9.21E-06	MF
GO:0043325	phosphatidylinositol-3,4-bispl	Myo1g;Ncf1	0.00102297	MF
GO:0005070	SH3/SH2 adaptor activity	Sh2d1a;Fcrl2	0.0013941	MF
GO:0005044	scavenger receptor activity	Fcrl2;Enpp2	0.00422599	MF
GO:0000166	nucleotide binding	Sult1a1;Chd1l	0.00511068	MF
GO:0005507	copper ion binding	Cp;Prnd	0.00548639	MF
GO:0016616	oxidoreductase activity, acting	Ldhc	0.0098713	MF
GO:0004551	nucleotide diphosphatase activ	Enpp2	0.0098713	MF
GO:0050294	steroid sulfotransferase activit	Sult1a1	0.0098713	MF
GO:0004062	aryl sulfotransferase activity	Sult1a1	0.0098713	MF
GO:0004322	ferroxidase activity	Cp	0.0098713	MF
GO:0030492	hemoglobin binding	Hbb	0.0098713	MF
GO:0047391	alkylglycerophosphoethanolar	Enpp2	0.01183418	MF
GO:0016176	superoxide-generating NADPI	Ncf1	0.01574865	MF
GO:0008020	G-protein coupled photorecep	Opn4	0.01574865	MF
GO:0004568	chitinase activity	Chi3l1	0.01574865	MF
GO:0016175	superoxide-generating NADPI	Ncf1	0.01574865	MF
GO:0015108	chloride transmembrane transp	Slc4a1	0.01770024	MF
GO:0008061	chitin binding	Chi3l1	0.01770024	MF
GO:0008510	sodium:bicarbonate symporter	Slc4a1	0.01770024	MF
GO:0008066	glutamate receptor activity	Grm4	0.01770024	MF
GO:0050662	coenzyme binding	Alas2	0.01964807	MF
GO:0001206	transcriptional repressor activi	Zbtb16	0.02159216	MF
GO:0005088	Ras guanyl-nucleotide exchan	Rasgrp3	0.02546914	MF
GO:0008195	phosphatidate phosphatase act	Lpin1	0.02546914	MF
GO:0015106	bicarbonate transmembrane tra	Slc4a1	0.02740203	MF
GO:0016594	glycine binding	Alas2	0.02933121	MF
GO:0030247	polysaccharide binding	Enpp2	0.03317844	MF
GO:0042975	peroxisome proliferator activa	Lpin1	0.03701088	MF
GO:0004622	lysophospholipase activity	Enpp2	0.03701088	MF
GO:0015301	anion:anion antiporter activity	Slc4a1	0.03892158	MF
GO:0005452	inorganic anion exchanger act	Slc4a1	0.03892158	MF
GO:0017016	Ras GTPase binding	Rasgrp3	0.04082859	MF
GO:0030506	ankyrin binding	Slc4a1	0.04082859	MF
GO:0004003	ATP-dependent DNA helicase	Chd1l	0.04273194	MF
GO:0030246	carbohydrate binding	Chi3l1;Ly49si2	0.04881282	MF
GO:0015671	oxygen transport	Hbe2;LOC689064;Hbb-b1;LC	6.40E-16	BP

GO:0048821	erythrocyte development	Hba1;Hbb;Bcl6;Hba-a2	1.26E-07	BP
GO:0042744	hydrogen peroxide catabolic p	Hba1;Hbb;Hba-a2	5.47E-06	BP
GO:0042542	response to hydrogen peroxide	Slc4a1;Hbb;Hba-a2;Hba1	1.74E-05	BP
GO:0006968	cellular defense response	Sh2d1a;Ncf1	0.00020201	BP
GO:0010942	positive regulation of cell deat	Hba1;Hbb;Hba-a2	0.00026258	BP
GO:0031065	positive regulation of histone	Lpin1;Bcl6	0.00043806	BP
GO:0001953	negative regulation of cell-ma	Bcl6;Enpp2	0.00049586	BP
GO:0051291	protein heterooligomerization	Hba1;Hbb;Hba-a2	0.00108805	BP
GO:0071276	cellular response to cadmium	Enpp2;Ncf1	0.00156097	BP
GO:0009967	positive regulation of signal tr	Sh2d1a;Fcrl2	0.00276494	BP
GO:0006879	cellular iron ion homeostasis	Cp;Alas2	0.00333701	BP
GO:0043087	regulation of GTPase activity	Rasgrp3;Bcl6	0.00715144	BP
GO:0009812	flavonoid metabolic process	Sult1a1	0.00974316	BP
GO:0014051	gamma-aminobutyric acid sec	Grm4	0.00974316	BP
GO:0016056	rhodopsin mediated signaling	Opn4	0.00974316	BP
GO:0072606	interleukin-8 secretion	Chi3l1	0.00974316	BP
GO:0045629	negative regulation of T-helpe	Bcl6	0.00974316	BP
GO:0006584	catecholamine metabolic proc	Sult1a1	0.00974316	BP
GO:0030097	hemopoiesis	Zbtb16;Hbb	0.01016261	BP
GO:0042541	hemoglobin biosynthetic proc	Alas2	0.01168071	BP
GO:0006642	triglyceride mobilization	Lpin1	0.01168071	BP
GO:0051138	positive regulation of NK T ce	Zbtb16	0.01168071	BP
GO:0071800	podosome assembly	Ncf1	0.01168071	BP
GO:0002467	germinal center formation	Bcl6	0.01361457	BP
GO:0070741	response to interleukin-6	Chi3l1	0.01361457	BP
GO:0038096	Fc-gamma receptor signaling	Myo1g	0.01361457	BP
GO:2000394	positive regulation of lamellip	Enpp2	0.01361457	BP
GO:0014823	response to activity	Sult1a1;Slc4a1	0.01539663	BP
GO:0045986	negative regulation of smooth	Ncf1	0.01554477	BP
GO:0006782	protoporphyrinogen IX biosyn	Alas2	0.01554477	BP
GO:0050427	3'-phosphoadenosine 5'-phosp	Sult1a1	0.01554477	BP
GO:0001878	response to yeast	Ncf1	0.01554477	BP
GO:0006691	leukotriene metabolic process	Ncf1	0.01554477	BP
GO:0006032	chitin catabolic process	Chi3l1	0.01554477	BP
GO:0019752	carboxylic acid metabolic proc	Ldhc	0.01554477	BP
GO:0072678	T cell migration	Myo1g	0.01554477	BP
GO:0002829	negative regulation of type 2 i	Bcl6	0.01554477	BP
GO:0002456	T cell mediated immunity	Myo1g	0.01554477	BP
GO:0007196	adenylate cyclase-inhibiting G	Grm4	0.01747129	BP
GO:0050665	hydrogen peroxide biosynthet	Ncf1	0.01747129	BP
GO:0006898	receptor-mediated endocytosis	Fcrl2;Enpp2	0.01788076	BP
GO:0045730	respiratory burst	Ncf1	0.01939416	BP
GO:0006090	pyruvate metabolic process	Ldhc	0.01939416	BP
GO:0051923	sulfation	Sult1a1	0.01939416	BP
GO:0018298	protein-chromophore linkage	Opn4	0.01939416	BP
GO:0006825	copper ion transport	Cp	0.01939416	BP
GO:0045741	positive regulation of epiderm	Ncf1	0.01939416	BP
GO:0005975	carbohydrate metabolic proces	Chi3l1;Ldhc	0.01984801	BP
GO:0032025	response to cobalt ion	Alas2	0.02131338	BP

GO:0008210	estrogen metabolic process	Sult1a1	0.02131338	BP
GO:0051272	positive regulation of cellular	Bcl6	0.02131338	BP
GO:0042554	superoxide anion generation	Ncf1	0.02131338	BP
GO:0050805	negative regulation of synapti	Grm4	0.02322895	BP
GO:0097484	dendrite extension	Cdkl3	0.02322895	BP
GO:2000773	negative regulation of cellular	Bcl6	0.02322895	BP
GO:0045591	positive regulation of regulato	Bcl6	0.02322895	BP
GO:0019432	triglyceride biosynthetic proce	Lpin1	0.02322895	BP
GO:0006878	cellular copper ion homeostas	Prnd	0.02322895	BP
GO:0009062	fatty acid catabolic process	Lpin1	0.02514087	BP
GO:0007340	acrosome reaction	Prnd	0.02704917	BP
GO:0006068	ethanol catabolic process	Sult1a1	0.02704917	BP
GO:0001823	mesonephros development	Zbtb16	0.02704917	BP
GO:0035137	hindlimb morphogenesis	Zbtb16	0.02704917	BP
GO:0031529	ruffle organization	Lpin1	0.02704917	BP
GO:0035136	forelimb morphogenesis	Zbtb16	0.02704917	BP
GO:0061036	positive regulation of cartilag	Zbtb16	0.02704917	BP
GO:0051932	synaptic transmission, GABA	Grm4	0.02704917	BP
GO:0006820	anion transport	Slc4a1	0.02704917	BP
GO:0030324	lung development	Cp;Chi3l1	0.02740296	BP
GO:0009414	response to water deprivation	Slc4a1	0.02895384	BP
GO:0010447	response to acidic pH	Slc4a1	0.0308549	BP
GO:0007602	phototransduction	Opn4	0.0308549	BP
GO:0007250	activation of NF-kappaB-indu	Chi3l1	0.0308549	BP
GO:0006754	ATP biosynthetic process	Ldhc	0.0308549	BP
GO:0017085	response to insecticide	Sult1a1	0.0308549	BP
GO:0035634	response to stilbenoid	Hba1	0.0308549	BP
GO:0006783	heme biosynthetic process	Alas2	0.0308549	BP
GO:0009395	phospholipid catabolic proces	Enpp2	0.03275233	BP
GO:0045598	regulation of fat cell differenti	Lpin1	0.03464617	BP
GO:0006801	superoxide metabolic process	Ncf1	0.03464617	BP
GO:0032332	positive regulation of chondro	Zbtb16	0.0365364	BP
GO:0031589	cell-substrate adhesion	Myo1g	0.0365364	BP
GO:0007283	spermatogenesis	Pcdha3;Bcl6;Prnd	0.03737964	BP
GO:0050832	defense response to fungus	Ncf1	0.03842304	BP
GO:0006612	protein targeting to membrane	Ncf1	0.03842304	BP
GO:0008285	negative regulation of cell pro	Zbtb16;Bcl6;Grm4	0.03964269	BP
GO:0048714	positive regulation of oligode	Enpp2	0.0403061	BP
GO:0046685	response to arsenic-containing	Slc4a1	0.0403061	BP
GO:0015698	inorganic anion transport	Slc4a1	0.0403061	BP
GO:1900745	positive regulation of p38MA	Ncf1	0.0403061	BP
GO:0045638	negative regulation of myeloic	Zbtb16	0.0403061	BP
GO:0035024	negative regulation of Rho pro	Bcl6	0.04218558	BP
GO:0045778	positive regulation of ossifica	Zbtb16	0.04218558	BP
GO:0000266	mitochondrial fission	Lpin1	0.04218558	BP
GO:0030517	negative regulation of axon ex	Cdkl3	0.04406148	BP
GO:0061003	positive regulation of dendriti	Bhlhb9	0.04593382	BP
GO:0015701	bicarbonate transport	Slc4a1	0.04593382	BP

GO:0051966	regulation of synaptic transmi	Grm4	0.0478026	BP

Term_ID	Term_description	GeneSymb	P_value	Category
GO:0014069	postsynaptic density	Palm;Dst;I	5.65E-05	CC
GO:0030018	Z disc	Cacna1c;S	0.001057	CC
GO:0009898	cytoplasmic side of pl	Palm;Dst	0.003534	CC
GO:1990454	L-type voltage-gated c	Cacna1c	0.011435	CC
GO:0005634	nucleus	Palm;Pbx2	0.012813	CC
GO:0036057	slit diaphragm	Nphs1	0.013058	CC
GO:0044309	neuron spine	Palm	0.013058	CC
GO:0097512	cardiac myofibril	Lmod2	0.014678	CC
GO:0034993	LINC complex	Syne3	0.014678	CC
GO:0030056	hemidesmosome	Dst	0.016296	CC
GO:0000791	euchromatin	Uhrf1	0.016296	CC
GO:0042383	sarcolemma	Cacna1c;D	0.017544	CC
GO:0005865	striated muscle thin fil	Lmod2	0.017912	CC
GO:0030061	mitochondrial crista	Akap1	0.019525	CC
GO:0032591	dendritic spine membr	Palm	0.019525	CC
GO:0097038	perinuclear endoplasm	Dst	0.024348	CC
GO:0005635	nuclear envelope	Syne3;Dst	0.02725	CC
GO:0031527	filopodium membrane	Palm	0.027551	CC
GO:0005665	DNA-directed RNA p	Znf768	0.029148	CC
GO:0035371	microtubule plus-end	Dst	0.032336	CC
GO:0005640	nuclear outer membra	Syne3	0.033926	CC
GO:0016327	apicolateral plasma m	Palm	0.033926	CC
GO:0043234	protein complex	Hira;Cacna	0.034446	CC
GO:0015629	actin cytoskeleton	Synpo2l;D	0.036099	CC
GO:0005657	replication fork	Uhrf1	0.038681	CC
GO:0005891	voltage-gated calcium	Cacna1c	0.038681	CC
GO:0031430	M band	Lmod2	0.044985	CC
GO:0005720	nuclear heterochromat	Uhrf1	0.044985	CC
GO:0032590	dendrite membrane	Palm	0.044985	CC
GO:0045211	postsynaptic membran	Cacna1c;A	0.046299	CC
GO:0051393	alpha-actinin binding	Cacna1c;N	0.000828	MF
GO:0019904	protein domain specifi	Akap1;Cac	0.00374	MF
GO:0008092	cytoskeletal protein bi	Dlg5;Syne	0.006294	MF
GO:0004843	thiol-dependent ubiqu	Tnfaip3;U	0.007907	MF
GO:0048248	CXCR3 chemokine re	Cxcl11	0.009275	MF
GO:0003779	actin binding	Synpo2l;D	0.009322	MF
GO:0061578	Lys63-specific deubiq	Tnfaip3	0.01112	MF
GO:0030346	protein phosphatase 2	Akap1	0.016635	MF
GO:0042379	chemokine receptor bi	Cxcl11	0.018467	MF
GO:0004143	diacylglycerol kinase	Dgkh	0.018467	MF
GO:0051010	microtubule plus-end	Dst	0.022121	MF
GO:0008331	high voltage-gated cal	Cacna1c	0.022121	MF
GO:0005272	sodium channel activi	Asic4	0.027577	MF
GO:0005523	tropomyosin binding	Lmod2	0.027577	MF
GO:0003714	transcription corepres	Hira;Zhx3	0.028946	MF
GO:0003951	NAD+ kinase activity	Dgkh	0.031197	MF
GO:0046872	metal ion binding	Tab3;Zsca	0.031672	MF
GO:0008327	methyl-CpG binding	Uhrf1	0.033003	MF
GO:0031491	nucleosome binding	Hira	0.036604	MF

GO:0003700	DNA binding transcri	Bcl3;Zhx3	0.037023	MF
GO:0003677	DNA binding	Zfp37;Tnf	0.03794	MF
GO:0034237	protein kinase A regul	Akap1	0.0384	MF
GO:0070530	K63-linked polyubiqu	Tnfaip3	0.040193	MF
GO:0031369	translation initiation f	Cacna1c	0.043768	MF
GO:0017022	myosin binding	Nphs1	0.04733	MF
GO:0000987	proximal promoter sec	Uhrf1	0.04733	MF
GO:0003785	actin monomer bindin	Lmod2	0.04733	MF
GO:0030011	maintenance of cell pc	Dlg5;Dst	4.30E-05	BP
GO:0007010	cytoskeleton organizat	Syne3;Palı	0.00125	BP
GO:0006351	transcription, DNA-te	Zscan10;H	0.001326	BP
GO:0060999	positive regulation of	Dlg5;Palm	0.001749	BP
GO:0030838	positive regulation of	Nphs1;Lm	0.00322	BP
GO:0070098	chemokine-mediated s	Cxcl11;Tff	0.004483	BP
GO:0016579	protein deubiquitinati	Tnfaip3;U	0.006646	BP
GO:0086045	membrane depolarizat	Cacna1c	0.008601	BP
GO:0042536	negative regulation of	Bcl3	0.008601	BP
GO:0050728	negative regulation of	Tff2;Tnfai	0.009836	BP
GO:0010216	maintenance of DNA	Uhrf1	0.010312	BP
GO:0060455	negative regulation of	Tff2	0.010312	BP
GO:0002455	humoral immune resp	Bcl3	0.010312	BP
GO:0051101	regulation of DNA bir	Bcl3	0.010312	BP
GO:0051534	negative regulation of	Akap1	0.010312	BP
GO:0043031	negative regulation of	Tff2	0.010312	BP
GO:0031581	hemidesmosome asser	Dst	0.010312	BP
GO:0007015	actin filament organiz	Sorbs2;Lm	0.010496	BP
GO:0050869	negative regulation of	Tnfaip3	0.012021	BP
GO:0072205	metanephric collecting	Dlg5	0.012021	BP
GO:0086012	membrane depolarizat	Cacna1c	0.012021	BP
GO:0002467	germinal center forma	Bcl3	0.012021	BP
GO:2000347	positive regulation of	Tnfaip3	0.012021	BP
GO:0030252	growth hormone secre	Cacna1c	0.012021	BP
GO:1900246	positive regulation of	Trim15	0.012021	BP
GO:0045088	regulation of innate in	Tnfaip3	0.013727	BP
GO:0051694	pointed-end actin filar	Lmod2	0.013727	BP
GO:0035331	negative regulation of	Dlg5	0.013727	BP
GO:0001505	regulation of neurotra	Celf6	0.013727	BP
GO:0090286	cytoskeletal anchoring	Syne3	0.013727	BP
GO:0045010	actin nucleation	Lmod2	0.013727	BP
GO:0032836	glomerular basement	Nphs1	0.01543	BP
GO:0046620	regulation of organ gr	Cacna1c	0.01543	BP
GO:0060074	synapse maturation	Palm	0.01543	BP
GO:0010818	T cell chemotaxis	Cxcl11	0.01543	BP
GO:0002315	marginal zone B cell c	Bcl3	0.01543	BP
GO:0045176	apical protein localiza	Dlg5	0.01713	BP
GO:0061337	cardiac conduction	Cacna1c	0.01713	BP
GO:0072015	glomerular visceral ep	Nphs1	0.01713	BP
GO:0042088	T-helper 1 type immu	Bcl3	0.01713	BP

GO:0035871	protein K11-linked de	Tnfaip3	0.01713	BP
GO:0031110	regulation of microtub	Dst	0.01713	BP
GO:0010225	response to UV-C	Bcl3	0.01713	BP
GO:0008090	retrograde axonal tran	Dst	0.018828	BP
GO:0010390	histone monoubiquitin	Uhrf1	0.018828	BP
GO:0002237	response to molecule (	Tnfaip3	0.018828	BP
GO:0035308	negative regulation of	Akap1	0.018828	BP
GO:0098911	regulation of ventricul	Cacna1c	0.018828	BP
GO:0032691	negative regulation of	Tnfaip3	0.018828	BP
GO:0008360	regulation of cell shap	Syne3;Palr	0.02016	BP
GO:1903364	positive regulation of	Tnfaip3	0.020522	BP
GO:0061049	cell growth involved i	Sorbs2	0.020522	BP
GO:0003094	glomerular filtration	Nphs1	0.020522	BP
GO:0002690	positive regulation of	Cxcl11	0.020522	BP
GO:0032495	response to muramyl d	Tnfaip3	0.020522	BP
GO:0090150	establishment of prote	Syne3	0.020522	BP
GO:0010614	negative regulation of	Akap1	0.020522	BP
GO:0032233	positive regulation of	Synpo2l	0.022214	BP
GO:0016574	histone ubiquitination	Uhrf1	0.022214	BP
GO:0045824	negative regulation of	Tnfaip3	0.022214	BP
GO:0016310	phosphorylation	Dgkh;Akap	0.023542	BP
GO:0043950	positive regulation of	Cxcl11	0.023903	BP
GO:0086002	cardiac muscle cell ac	Cacna1c	0.023903	BP
GO:0071625	vocalization behavior	Celf6	0.023903	BP
GO:0030330	DNA damage respons	Bcl3	0.023903	BP
GO:0045104	intermediate filament	Dst	0.025589	BP
GO:0045197	establishment or main	Dlg5	0.025589	BP
GO:1902187	negative regulation of	Trim15	0.025589	BP
GO:0030816	positive regulation of	Cxcl11	0.027272	BP
GO:0006939	smooth muscle contra	Cacna1c	0.027272	BP
GO:0010738	regulation of protein k	Akap1	0.027272	BP
GO:0032703	negative regulation of	Tnfaip3	0.027272	BP
GO:0032481	positive regulation of	Trim15	0.028952	BP
GO:0032270	positive regulation of	Uhrf1	0.028952	BP
GO:0019229	regulation of vasocons	Cacna1c	0.028952	BP
GO:0035418	protein localization to	Nphs1	0.028952	BP
GO:0048870	cell motility	Dst	0.03063	BP
GO:0051056	regulation of small GT	Arhgap26	0.03063	BP
GO:0017156	calcium ion regulated	Cacna1c	0.03063	BP
GO:0042692	muscle cell differentia	Hira	0.03063	BP
GO:0071257	cellular response to el	Palm	0.03063	BP
GO:1903507	negative regulation of	Hira	0.033977	BP
GO:0030818	negative regulation of	Palm	0.033977	BP
GO:0070536	protein K63-linked de	Tnfaip3	0.035646	BP
GO:0046907	intracellular transport	Dst	0.037312	BP
GO:0007520	myoblast fusion	Nphs1	0.037312	BP
GO:0045736	negative regulation of	Tnfaip3	0.037312	BP
GO:0070509	calcium ion import	Cacna1c	0.038976	BP

GO:0006336	DNA replication-inde	Hira	0.038976	BP
GO:0060441	epithelial tube branchi	Dlg5	0.040637	BP
GO:0030041	actin filament polyme	Lmod2	0.040637	BP
GO:1902042	negative regulation of	Tnfaip3	0.042295	BP
GO:0007194	negative regulation of	Palm	0.042295	BP
GO:0071108	protein K48-linked de	Tnfaip3	0.042295	BP
GO:0009954	proximal/distal patter	Pbx2	0.04395	BP
GO:0042832	defense response to pr	Bcl3	0.047252	BP
GO:0007205	protein kinase C-activ	Dgkh	0.047252	BP
GO:0007586	digestion	Tff2	0.047252	BP
GO:2000352	negative regulation of	Tnfaip3	0.048899	BP
